# What belongs together goes together: the speaker-hearer perspective. A commentary on MacDonald's PDC account

**DOI:** 10.3389/fpsyg.2013.00228

**Published:** 2013-05-03

**Authors:** Peter Hagoort, Antje S. Meyer

**Affiliations:** Max Planck Institute for PsycholinguisticsNijmegen, Netherlands

MacDonald (2013) proposes that distributional properties of language and processing biases in language comprehension can to a large extent be attributed to consequences of the language production process. In essence, the account is derived from the principle of least effort that was formulated by Zipf, among others (Zipf, 1949; Levelt, 2013). However, in Zipf's view the outcome of the least effort principle was a compromise between least effort for the speaker and least effort for the listener, whereas MacDonald puts most of the burden on the production process.

There is much in this paper that we whole-heartedly agree with. Aiming to link language change and typology to specific, independently established cognitive principles is a timely and exciting research program. We are pleased to see speakers and their needs take a central position in this enterprise, and relative clauses be demoted from their key position in psycholinguistic research.

Although we are quite sympathetic to the proposed program and the least effort principle as determining the statistical regularities in the linguistic input, we believe that MacDonald's account has two shortcomings. One is that the human language user is sketched as someone who can be in the state of “pure production,” while in fact she is inherently a speaker-hearer, so that it is impossible to segregate production and comprehension in any strict sense. The second shortcoming is that the PDC account is mostly based on the production of single sentences, something that hardly ever happens in daily language use. We will discuss the consequences of both observations about language users for the PDC account.

The distinction between the roles of speakers and listeners in conversation is sensible and easy to make—speakers talk and listeners listen. It is much harder to distinguish the cognitive processes underlying speaking and listening. Are there, apart from overt articulation, any processes that are only involved in speaking but not in listening? And are there any processes that are exclusively involved in listening but not in speaking? However, in our view there is to date no convincing evidence for the existence of components of linguistic knowledge that would be exclusively recruited during speaking or exclusively during listening. Similarly, there is no evidence for access or encoding processes that would occur only during speaking or during listening. By contrast, there are good theoretical arguments and strong empirical evidence for the opposite, more parsimonious view that the knowledge structures and access processes involved in speaking and listening are largely shared. This point has, of course, been made in many contexts. We just provide a few examples. (i) Models of lexical access often assume shared semantic representations and either shared word form representations or tightly linked input and output forms (e.g., Levelt et al., 1999; Goldrick and Rapp, 2006); (ii) Theories of self-monitoring typically assume that monitoring during speaking involves the speech comprehension system, with speakers processing their phonological utterance plans as they would process external speech input (e.g., Levelt, 1989); or they assume monitoring processes that are internal to a speech planning system that is shared with the comprehension system (e.g., Slevc and Ferreira, 2006); (iii) Grammatical encoding in production and comprehension has been argued to involve shared routines (Kempen et al., 2012) and neural substrates. Using a repetition suppression paradigm recent studies (Menenti et al., 2011; Segaert et al., 2012) have shown that syntactic priming results in repetition suppression in the same brain areas for production and comprehension but also between production and comprehension. These results provide clear evidence that syntactic encoding and parsing share neural infrastructure. Or in terms of the PDC account, Plan Reuse is shared between production and comprehension; (iv) The assumption that listening and speaking involve shared knowledge structures and access routines is the foundation of Pickering and Garrod's (2004) alignment theory of dialog; (v) Finally, several authors have stressed the role of prediction of upcoming utterance parts in comprehension, at all levels of processing, from semantics to phonetics. Listeners are known to predict upcoming words not only conceptually, but also in terms of their syntactic (Van Berkum et al., 2005) and phonological (DeLong et al., 2005) make-up. The most parsimonious explanation is that prediction uses the production route. Prediction is production. Hence for many utterances that we comprehend production might be at stake. Thus, the speaker is a hearer, and the hearer is sometimes a (silent) speaker. In short, speaking and listening are highly intertwined processes. Therefore, it will be almost impossible to know if the least effort is driven by production or comprehension since at multiple levels they go together necessarily.

One might say that even if the processes involved in speaking and listening are largely the same, the processing principles are primarily brought to bear during speaking, rather than listening. This is because they concern how conceptual and linguistic information is linearized, and linearization is the speakers' task; the listener simply has to cope with the information in whatever order it is presented. However, if shared processes and knowledge structures are involved in speaking and listening, principles that facilitate speech planning should also facilitate the comprehension of utterances. For instance, the Easy First principle may be a good principle for planning complex utterances, but adhering to this principle may also facilitate comprehension because the listener expects the speaker to adhere to this principle. Thus, even though the speaker takes control in linearizing utterances, the listener has to play along, and usually can easily do so, precisely because speaking and listening are tightly related. Hence, whether learning occurs primarily through speaking or through listening is by no means clear (Chang et al., 2006).

A second important fact is that language comprehension and production hardly ever happen by interpreting or producing single utterances, but almost always in a conversational setting or embedded in a wider discourse. Discourse and conversational settings are organized according to the principles of information structure. Topic and comment specify what is the background information and what is new (Seuren, 2009). These factors co-determine the ordering of information, lexical choice, and syntactic structure. For instance, following the question “The meat was bought by whom?” the expected form of the produced utterance will be “The meat was bought by X.” Despite single sentence statistics favoring animate subjects and active sentence structure, these biases are completely overwritten by the information structure requirements in a multi-utterance discourse. In that sense, one cannot put word length and frequency on a par with givenness as is done by MacDonald. Givenness is determined by discourse context and/or the utterance of the conversational partner and hence dependent on comprehension. Computational limitations are not only dependent on long-term memory structures, but also on current context, or in other words on a situation model built up through the comprehension route. The least effort for the speaker is a joint product of the ease of retrieval of information laid down in memory by many previous instances, modulated by what the current context provides in aiding retrieval of utterance plans and word information. In this way comprehension co-determines the priors for the to-be-produced utterance.

The blind spot that we see in MacDonald's account is presumably related to the analogy between language production and action planning that she makes. However, since dialog and conversation are the most natural ways of language use, the default planning is not one of action but of *joined* action. That is, planning an utterance is guided by and occurs in the context of comprehending the other's utterance and inferring her intention. In a joined action perspective it is less clear how one can isolate production factors from contributions of the comprehension process.

A final comment concerns the nature of the three key processing principles discussed in the paper—Easy First, Plan Reuse, Avoid Interference. As the author stresses throughout the paper, the principles are not specific to speaking (or listening). Instead they are domain-general and are applied in many cognitive tasks, including speaking. Thus, the proposal is not about the way language production shapes language form and comprehension, but about the way cognition shapes language production, language form, and comprehension.

In summary, we agree that many distributional patterns in language and multiple phenomena in language processing can be explained in terms of the principle of least effort. However, we think that language production and comprehension are so clearly interwoven at multiple levels that it makes no sense to attribute the consequences almost exclusively to one side of the speaker-hearer.

## References

[B1] Chang F. Dell G. S. Bock K. (2006). Becoming syntactic. Psychol. Rev. 113, 234–272 10.1037/0033-295X.113.2.234 16637761

[B2] DeLong K. A. Urbach T. P. Kutas M. (2005). Probabilistic word pre-activation during language comprehension inferred from electrical brain activity. Nat. Neurosci. 8, 1117–1121 10.1038/nn1504 16007080

[B3] Goldrick M. Rapp B. (2006). Lexical and post-lexical representations in spoken production. Cognition 102, 219–260 10.1016/j.cognition.2005.12.010 16483561

[B4] Kempen G. Olsthoorn N. Sprenger S. (2012). Grammatical workspace sharing during language production and language comprehension: evidence from grammatical multitasking. Lang. Cogn. Process. 27, 345–380

[B6a] Levelt W. J. M. (1989). Speaking: From Intention to Articulation. Cambridge, MA: MIT press

[B5] Levelt W. J. M. (2013). A History of Psycholinguistics. The Pre-Chomskyan Era. Oxford: Oxford University Press

[B6] Levelt W. J. M. Roelofs A. Meyer A. S. (1999). A theory of lexical access in speech production. Behav. Brain Sci. 22, 1–38 11301520 10.1017/s0140525x99001776

[B13a] MacDonald M. C. (2013). How language production shapes language form and comprehension. Front. Psychol. 4:226 10.3389/fpsyg.2013.00226 PMC3636467 23637689

[B7] Menenti L. Gierhan S. Segaert K. Hagoort P. (2011). Shared language: overlap and segregation of the neuronal infrastructure for speaking and listening revealed by functional MRI. Psychol. Sci. 22, 1173–1182 10.1177/0956797611418347 21841148

[B8] Pickering M. J. Garrod S. (2004). Toward a mechanistic psychology of dialogue. Behav. Brain Sci. 27, 169 15595235 10.1017/s0140525x04000056

[B9] Segaert K. Menenti L. Weber K. Petersson K. M. Hagoort P. (2012). Shared syntax in language production and language comprehension – an fMRI study. Cereb. Cortex 22, 1662–1670 10.1093/cercor/bhr249 21934094 PMC3377967

[B10] Seuren P. A. M. (2009). Language in Cognition. Language From Within, Vol. 1. Oxford: Oxford University Press

[B11] Slevc L. R. Ferreira V. S. (2006). Halting in single word production: a test of the perceptual loop theory of speech monitoring. J. Mem. Lang. 54, 515–540 10.1016/j.jml.2005.11.002 17917694 PMC2000858

[B12] Van Berkum J. J. A. Brown C. M. Zwitserlood P. Kooijman V. Hagoort P. (2005). Anticipating upcoming words in discourse: evidence from ERPs and reading times. J. Exp. Psychol. Learn. Mem. Cogn. 31, 443–467 10.1037/0278-7393.31.3.443 15910130

[B13] Zipf G. K. (1949). Human Behavior and the Principle of Least Effort. Cambridge, MA: Addison-Wesley Press

